# The Relationship between Dietary Patterns and Metabolic Health in a Representative Sample of Adult Australians

**DOI:** 10.3390/nu7085295

**Published:** 2015-08-05

**Authors:** Lucinda K. Bell, Suzanne Edwards, Jessica A. Grieger

**Affiliations:** 1 Nutrition and Dietetics, School of Health Sciences, Faculty of Medicine, Nursing and Health Sciences, Flinders University, Bedford Park 5042, Australia; E-Mail: lucy.bell@flinders.edu.au; 2 Data Management and Analysis Centre (DMAC), Faculty of Health Sciences, University of Adelaide, Adelaide 5005, Australia; E-Mail: suzanne.edwards@adelaide.edu.au; 3 Robinson Research Institute, School of Medicine, Faculty of Health Sciences, University of Adelaide, Adelaide 5005, Australia

**Keywords:** dietary patterns, metabolic health, obesity, Australia, national survey, body mass index, adults

## Abstract

Studies assessing dietary intake and its relationship to metabolic phenotype are emerging, but limited. The aims of the study are to identify dietary patterns in Australian adults, and to determine whether these dietary patterns are associated with metabolic phenotype and obesity. Cross-sectional data from the Australian Bureau of Statistics 2011 Australian Health Survey was analysed. Subjects included adults aged 45 years and over (*n* = 2415). Metabolic phenotype was determined according to criteria used to define metabolic syndrome (0–2 abnormalities *vs.* 3–7 abnormalities), and additionally categorized for obesity (body mass index (BMI) ≥30 kg/m^2^ *vs.* BMI <30 kg/m^2^). Dietary patterns were derived using factor analysis. Multivariable models were used to assess the relationship between dietary patterns and metabolic phenotype, with adjustment for age, sex, smoking status, socio-economic indexes for areas, physical activity and daily energy intake. Twenty percent of the population was metabolically unhealthy and obese. In the fully adjusted model, for every one standard deviation increase in the Healthy dietary pattern, the odds of having a more metabolically healthy profile increased by 16% (odds ratio (OR) 1.16; 95% confidence interval (CI): 1.04, 1.29). Poor metabolic profile and obesity are prevalent in Australian adults and a healthier dietary pattern plays a role in a metabolic and BMI phenotypes. Nutritional strategies addressing metabolic syndrome criteria and targeting obesity are recommended in order to improve metabolic phenotype and potential disease burden.

## 1. Introduction

Metabolic abnormalities such as insulin resistance, hypertension, dyslipidemia and abnormal glucose metabolism place individuals at increased risk of cardiovascular diseases (CVD), type 2 diabetes and mortality [1,2,3]. Such abnormalities are commonly associated with obesity [4], yet there are a proportion of obese individuals (approximately 10%–25%) [5] that have a normal metabolic profile [5,6]. These individuals are termed “metabolically healthy and obese”. Similarly, some normal weight individuals display an abnormal metabolic profile typically seen in obese individuals and are therefore termed “metabolically unhealthy, not obese”. These phenotypes may carry varying disease and mortality risk [4,7].

The dietary determinants underlying metabolic health are not fully understood. Epidemiological studies that have focused on the role of single foods and/or individual nutrients have produced inconclusive findings regarding their influence on overall (not individual markers of) metabolic health [3,8,9]. This may be due to the complex nature of diet and the synergies between dietary constituents [10,11]. Thus, dietary patterns may be a more useful means for understanding the influence of diet on metabolic health as they examine the total effect of food and nutrient combinations and are reflective of the way people eat, that is, consumption of foods or meals rather than individual dietary constituents [10,11]. One recent review highlighted the beneficial effects of foods and nutrients contained in the Mediterranean dietary pattern, the DASH (dietary approaches to stop hypertension) diet, and the Nordic diet (based on traditional foods consumed in Northern Europe), including fruits, vegetables, wholegrains, dairy, vitamin D, calcium and omega 3 fatty acids on individual markers of metabolic syndrome [12]. However, the clustering of metabolic components associated with each dietary pattern was not quantitatively assessed. Nevertheless, despite a growing body of evidence investigating and demonstrating an association between dietary patterns and metabolic abnormalities, to our knowledge, none have been conducted in Australian adults. Further, the relationship between dietary patterns and varying metabolic phenotype has rarely been investigated, with most assessing metabolic health in general, and not accounting for obesity. Whether dietary patterns are associated with varying metabolic phenotypes within the context of an obese or non-obese body mass index (BMI) in Australian adults is unknown.

Therefore, the aims of this study are: (1) to identify dietary patterns in Australian adults aged ≥ 45 years; and (2) to determine whether the dietary patterns identified are associated with metabolic phenotype in those who are obese and non-obese.

## 2. Experimental Section

### 2.1. Data and Study Population

Data was from the Australian Bureau of Statistics (ABS) Confidentialised Unit Record Files (CURF) obtained in the 2011–2013 Australian Health Survey (AHS) with access to the data using the Remote Access Data Laboratory. A full description of the methods for data collection in the AHS has been reported by the ABS [13]. Within the confidentialised unit record files, survey data was collected using the *National Health Survey* (NHS), the *National Nutrition and Physical Activity Survey* (NNPAS), and the *National Health Measures Survey* (NHMS), which included a biomedical component. Both the NHS and the NNPAS were conducted using a stratified multistage area sample of private dwellings, with participants aged 2 years and over. In the NHS, 21,108 private dwellings were selected (reduced to an actual sample of 18,355 dwellings after sample loss in the field stage), in which 84.8% were fully or adequately responding households (*n* = 15,565). In the NNPAS, a total of 14,363 private dwellings were selected in the sample for the NNPAS (reduced to an actual sample of 12,366 dwellings after sample loss in the field stage), in which 77.0% were fully or adequately responding households to the first interview (*n* = 9519). Of the 30,329 respondents aged 5 years and over in the combined sample (NHS + NNPAS), 11,246 (37.1%) participated in the biomedical component (NHMS). The 2011–2012 NHS and NNPAS utilised Computer Assisted Interview instruments to collect the data [13].

Variables drawn from the datasets and included in this paper were age, sex, smoking status (categorized by the ABS as current smoker, never a smoker and previous/episodic smoker), Socio-Economic Indexes for Areas (SEIFA) derived from SEIFA deciles provided by the ABS 2011–2013 AHS, and physical activity (using the three categories provided by the ABS 2011–2013 AHS: inactive in last week, insufficiently active for health in last week, or sufficiently active for health in last week). Waist circumference and blood pressure data measured in the AHS were also used in the metabolic health definition (see below). Further details of types of data collection obtained for each survey can be found on the ABS website [13]. Adults aged 45 years and over and who had blood results recorded (at least total cholesterol) and who had the first 24-h recall completed, as this is most representative of the Australian population, were used in the current analysis (*n* = 2415).

### 2.2. Dietary Data

The 2011–2012 NNPAS collected dietary data that included: 24-h dietary recall of food, beverages, and supplements (on two separate days); usual dietary behaviours; and whether currently on a diet and for what reason. Briefly, the 24-h dietary recall questionnaire collected detailed information on all foods and beverages consumed on the day prior to interview. Where possible, at least eight days after the first interview, respondents were contacted to participate in a second 24-h dietary recall via telephone interview. The Automated Multiple-Pass Method was used to gather food intake data, where an automated questionnaire guides the interviewer through a system designed to maximise respondents’ opportunities for remembering and reporting foods eaten in the previous 24 h. Interviewers also used a Food Model Booklet to assist respondents with describing the amount of food and beverages consumed. The 24-h recall data was coded using the United States Department of Agriculture Dietary Intake Data System [14]. To allow for the coding of foods and measures, and the calculation of nutrients, Food Standards Australia and New Zealand developed a food and measures database. The database contains 5644 foods and 15,847 measures in which each food within the food database has a name, associated food description, inclusions, exclusions, and an eight-digit code. The eight-digit food codes are grouped into broader food groups (2-, 3- and 5- digit levels) based on groupings used in 1995 National Nutrition Survey. For the purpose of the analysis in this study, only the first 24-h recall was used (*n* = 2415 (100%) of participants; *n* = 1883 (78%) had 2 × 24-h recalls) and the minor food group categories (*i.e.*, 5-digit level) were coded into 39 food groups (Supplementary Table S1) for use in the factor analysis (see below).

### 2.3. Biomedical Measures

Key biomarkers measured in the NHMS included chronic disease biomarkers, tests for diabetes, cholesterol, triglycerides, kidney disease and liver function; as well as nutrient biomarkers (iron, folate, iodine and vitamin D). For the current analysis, biomarkers for “metabolic health” were: total cholesterol, high density lipoprotein cholesterol (HDL-C), low density lipoprotein cholesterol (LDL-C), triglycerides, and glucose. All of these measurements were obtained from a blood sample in those 12 years and over, in which LDL-C, triglycerides and glucose were taken in the fasted state.

### 2.4. Metabolic Health

Metabolic health was defined as follows: First, the following measures were dichotomised into “normal” and “abnormal” categories, where abnormal was defined as: (i) total cholesterol ≥ 5.5 mmol/L or having current diagnosis of high cholesterol; (ii) fasting LDL-C ≥ 3.5 mmol/L; (iii) HDL-C (accounting for sex) < 1.0 mmol/L for males and <1.3 mmol/L for females; (iv) fasting triglycerides ≥ 2.0 mmol/L; (v) fasting plasma glucose status > 6.0 mmol/L or having current diagnosis of type 2 diabetes or unknown type; (vi) waist circumference in males ≥102 cm or in females ≥88 cm; (vii) systolic blood pressure ≥ 140 mmHg or diastolic blood pressure ≥ 90 mmHg or having current diagnosis of hypertension. Second, based on the above “abnormal” categories, four categories relevant to metabolic health were created. That is, metabolically healthy, not obese (best outcome: 0–2 abnormal criteria and a BMI < 30 kg/m^2^) [15]; metabolically unhealthy, not obese (3–7 abnormal criteria and a BMI < 30 kg/m^2^); metabolically healthy and obese (0–2 abnormal criteria and a BMI ≥ 30 kg/m^2^); and metabolically unhealthy, and obese (poorest outcome: 3–7 abnormal criteria and a BMI ≥ 30 kg/m^2^). Missing values were reported for: LDL-C (*n* = 394, 16%), triglycerides (*n* = 366, 15%), fasting plasma glucose (*n* = 366, 15%), waist circumference (*n* = 97, 4%), and blood pressure (*n* = 87, 3.6%); variables with no missing data included total cholesterol, HDL-C, doctor-diagnosed high cholesterol, doctor-diagnosed diabetes, and doctor-diagnosed hypertension (*n* = 2415). Where there were missing values, the metabolic category (*i.e.*, normal/abnormal) for that measurement (e.g., LDL-C) was coded as “normal”.

### 2.5. Factor Analysis

Dietary patterns were derived using factor analysis with factor loadings extracted using the principal component method and varimax/orthogonal rotation. The number of dietary patterns identified was based on eigenvalues > 1.5, on identification of a break point in the scree plot, and on interpretability [16]. Using these criteria, a three-factor solution was chosen and rerun with the resulting factor scores saved and converted to *z*-scores for analysis. Items with factor loadings > 0.25 were considered as items of relevance for the identified factor. These items represent the foods most highly related to the identified factor [17]. Foods that cross-loaded on several factors were retained. Inter-item reliability for each factor was assessed using Cronbach’s α coefficients.

### 2.6. Statistical Analyses

Frequencies and descriptive data were assessed as *n* (%) or mean (standard deviation, SD). Ordinal logistic regression analysis (*i.e.*, 1. metabolically healthy, not obese (best outcome); 2. metabolically unhealthy, not obese; 3. metabolically healthy, and obese; and 4. metabolically unhealthy and obese (poorest outcome)) was used to determine the association between the dietary patterns and metabolic health, after adjusting for dietary energy intake (Model 1) and further adjusting for age, sex, smoking status, Socio-Economic Indexes for Areas (SEIFA) quintile, and physical activity (Model 2). The proportional odds assumption was tested and appeared reasonable. Multivariable linear regression was performed to investigate the association between number of metabolic abnormalities and the dietary patterns, also with adjustments for age, sex, smoking status, SEIFA quintile, physical activity, and daily energy intake. Population weights were applied (supplied by the ABS) to produce estimates at the population level using the “surveyreg” and “surveylogistic” procedures in SAS. The statistical software used within the Remote Access Data Laboratory was SAS 9.1 (SAS Institute Inc., Cary, NC, USA).

## 3. Results

Characteristics of the 2415 adults included in the analysis are reported in Table 1. The mean number of metabolic abnormalities was 2.2 and the median was 2.0. In the sample, 12% were metabolically healthy and obese; 48% were metabolically healthy, not obese; 20% were metabolically unhealthy, and obese; and 20% were metabolically unhealthy, not obese. In males and females combined, the most prevalent metabolic abnormality was high waist circumference (48%), followed by high LDL-C (43%), high total cholesterol (42%), high blood pressure (38%), low HDL-C (22%), high triglycerides (17%), and high plasma glucose (7%).

### 3.1. Dietary Patterns

Three dietary patterns were identified (Table 2). The variance explained by each factor was 9.8%, 7.5%, and 4.6%, respectively. Factor 1 was labelled Red meat and vegetable as red meat and several types of vegetables loaded on this pattern. Factor 2 was labelled Refined and processed as added sugar, full fat dairy, unsaturated spreads, cakes, pastries, and processed meat loaded on this pattern, while fresh fruit and vegetables were inversely correlated. Factor 3 was labelled Healthy as wholegrains, fresh fruit, dried fruit, legumes and low fat dairy loaded on this pattern, while take-away foods, soft drinks, alcoholic drinks, and fried potato were inversely correlated.

**Table 1:** Descriptive characteristics of the Australian sample aged 45 years and over, participating in the 2011–2013 Australian Health Survey.

		Red Meat and Vegetable			Refined and Processed			Healthy		
	All (*n* = 2415)	Tertile 1	Tertile 2	Tertile 3	Tertile 1	Tertile 2	Tertile 3	Tertile 1	Tertile 2	Tertile 3
Males/females (%)	48/52	49/51	43/57	53/47	37/63	43/57	66/34	53/47	43/57	50/50
Age group (%)										
45–60 years	54	60	50	52	53	55	53	57	54	50
61–70 years	25	20	27	27	27	22	24	26	23	25
>70 years	21	20	23	21	20	22	22	17	23	25
BMI (%)										
Obese (≥30 kg/m^2^)	31	40	32	32	27	34	33	33	33	28
Overweight (25–29.99 kg/m^2^)	40	30	39	39	43	38	38	44	35	40
Normal/underweight (<25 kg/m^2^)	29	30	29	29	31	28	29	23	32	32
Metabolic abnormalities (%)										
0	10	13	12	11	14	10	12	10	14	12
1–2	49	50	49	48	48	52	48	49	49	51
3–5	40	36	38	39	37	37	39	40	36	36
6–7	1	2	1	1	1	1	1	1	2	1
Physical activity (%)										
Inactive	22	25	17	24	17	24	25	22	26	17
Insufficiently active	26	23	31	25	24	24	32	27	24	29
Sufficiently active for health	52	52	51	51	59	52	43	51	50	54
SEIFA quintile (%)										
Quintile 1 (lowest)	19	17	20	18	17	18	21	19	18	20
Quintile 2	19	21	18	19	16	21	20	21	19	17
Quintile 3	21	22	19	22	19	20	23	19	22	20
Quintile 4	19	19	19	18	22	17	17	19	17	20
Quintile 5 (highest)	23	21	24	23	26	24	19	22	23	22
Smoking status (%)										
Current smoker	10	11	11	9	9	9	12	14	11	5
Never a smoker	47	46	47	48	50	47	43	39	47	54
Previous/episodic smoker	43	44	42	44	41	44	45	47	42	41

BMI: body mass index; SEIFA: Socio-Economic indexes for areas.

**Table 2:** Dietary patterns of the adults aged 45 years and over participating in the 2011–2013 Australian Health Survey.

Red Meat and Vegetable		Refined and Processed		Healthy	
Food Group	Factor Loading	Food Group	Factor Loading	Food Group	Factor Loading
Yellow or red vegetables	0.59	Added sugar	0.56	Whole grains	0.36
Potatoes	0.57	Full-fat dairy products	0.41	Fresh fruit	0.35
Red meats	0.50	Unsaturated spreads	0.36	Low-fat dairy products	0.33
Other vegetables	0.33	Cakes, biscuits, sweet pastries	0.32	Dried fruit	0.32
Cruciferous vegetables	0.29	Processed meat	0.25	Legumes	0.29
		Canned fruit	0.25	Unsaturated spreads	0.25
		Soft drinks	0.25		
Meat-based mixed dishes	−0.40	Other vegetables	−0.26	Take-away foods	−0.28
		Fresh fruit	−0.32	Soft drinks	−0.33
				Alcoholic drinks	−0.40
				Fried potatoes	−0.42

### 3.2. Dietary Patterns and Metabolic Health

In the ordinal logistic regression analysis, after adjustment for energy intake, there was a significant association between the four metabolic profile categories and the Refined and processed dietary pattern, such that for every one standard deviation increase in the Refined and processed pattern, the odds of having a more metabolically healthy profile decreased by 14% (odds ratio (OR) 0.86, 95% confidence interval (CI): 0.76, 0.98), while for every one standard deviation increase in the Healthy dietary pattern, the odds of having a more metabolically healthy profile increased by 18% (OR 1.18, 95% CI: 1.06, 1.31). In the fully adjusted models, only the association between the Healthy dietary pattern and a healthier metabolic profile remained significant (Table 3).

**Table 3:** Odds ratios (95% confidence interval (CI)) for metabolic profile ^1^, according to dietary pattern.

Metabolic Profile	Model 1 ^2^	95% CI	Model 2 ^3^	95% CI
Red meat and vegetable	0.97	0.88, 1.08	0.99	0.89, 1.10
Refined and processed	0.86	0.76, 0.98 *	0.92	0.81, 1.04
Healthy	1.18	1.06, 1.31 †	1.16	1.04, 1.29 †

^1^ Metabolic profile (ordinal logistic regression analysis with outcomes: 1. metabolically healthy, not obese (best outcome); 2. metabolically unhealthy, not obese; 3. metabolically healthy, and obese; and 4. metabolically unhealthy and obese (poorest outcome). Probabilities modelled are cumulated over the lower ordered values and metabolically healthy, not obese, is the lowest of the lower ordered values). ^2^ Adjusted for energy intake (energy *p* = 0.0728). ^3^Adjusted for energy intake, age (45–60; 61–70; > 70), sex, smoking status (current smoker, never a smoker, previous/episodic smoker), socio-economic indexes for areas quintile, physical activity level (inactive, insufficiently active, sufficiently active). * *p* < 0.05; † *p* < 0.01.

### 3.3. Dietary Patterns and Metabolic Abnormalities

In linear regression, a significant association was found between number of metabolic abnormalities and the Refined and processed dietary pattern (Model 1, *p* = 0.0089), such that for every one standard deviation increase in the Refined and processed pattern, the average number of metabolic abnormalities increases by 0.104 (Table 4). In the adjusted model however, the association did not reach statistical significance. In the unadjusted and adjusted analyses, there was no association for any of the dietary patterns and being metabolically healthy (0–2 abnormalities) or metabolically unhealthy (3–7 abnormalities) (Table 5).

**Table 4:** Linear regression estimates for number of metabolic abnormalities, according to dietary pattern.

Metabolic Number	Model 1 Estimate ^1^	95% CI	Model 2 Adjusted Estimate ^2^	95% CI
Red meat and vegetable	0.0007	−0.07, 0.07	−0.004	−0.07, 0.07
Refined and processed	0.10	0.03, 0.18	0.07	−0.01, 0.15
Healthy	−0.06	−0.13, 0.01	−0.04	−0.11, 0.03

^1^ Adjusted for energy intake (*p* = 0.0109); ^2^ Adjusted for energy intake, age (45–60; 61–70; > 70), sex, smoking status (current smoker, never a smoker, previous/episodic smoker), socio-economic indexes for areas quintile, physical activity level (inactive, insufficiently active, sufficiently active); CI, confidence interval.

**Table 5:** Odds ratios for metabolic health ^1^, according to dietary pattern.

Metabolic Health	Model 1 ^2^	95% CI	Model 2 ^3^	95% CI
Red meat and vegetable	1.00	0.90, 1.11	1.01	0.91, 1.12
Refined and processed	0.89	0.78, 1.02	0.94	0.82, 1.07
Healthy	1.10	0.98, 1.23	1.08	0.96, 1.21

^1^ Metabolically healthy (0–2 abnormalities) *vs.* those who are metabolically unhealthy (3–7 abnormalities); ^2^ Adjusted for energy intake (not significant); ^3^ Adjusted for energy intake, age (45–60; 61–70; >70), sex, smoking status (current smoker, never a smoker, previous/episodic smoker), socio-economic indexes for areas quintile, physical activity level (inactive, insufficiently active, sufficiently active); CI, confidence interval.

## 4. Discussion

To our knowledge, this is the first study reporting on the prevalence of metabolic health in a sample of adult Australians, and identifying associations between metabolic phenotype and current dietary patterns. We report that nearly half of the sample had a metabolically healthy profile (*i.e.*, metabolically healthy and not obese), while 40% of the remaining sample was metabolically unhealthy (regardless of BMI), and 12% were metabolically healthy and obese. The prevalence of the latter phenotype identified in this population is intermediate compared to 19% of adults (*n* = 4541) from the Atherosclerosis Risk in Communities study [18]; 8.2% reported in Thai men and women aged 18–59 years [19]; while in a sample of 780 women, 28% of women with normal BMI were metabolically unhealthy [20]. Further, in a population-based sample of 2803 women and 2557 men from Switzerland, six different criteria were used to define metabolically healthy obesity, with prevalence rates varying between 3.3% and 32.1% in men and between 11.4% and 43.3% in women [21]. Although differences in metabolic health criteria exist between studies, the fact that nearly half of our population was metabolically unhealthy is of concern as the biomarkers used to define metabolic health can be modified, particularly through diet and lifestyle changes. Irrespective of metabolic health, 71% of our population was either overweight or obese, which, in addition to poor metabolic health, has been consistently shown to play a key role in several chronic diseases [4,22].

Three dietary patterns were identified in the dietary pattern analysis. The dietary patterns resemble other dietary patterns, common in previous studies such as a prudent diet, similar to our red meat and vegetable pattern, a western diet, similar to our refined and processed pattern, and a healthy diet, comparable to our healthy pattern. After adjusting for energy intake only, we found that the odds for having a more metabolically healthy profile decreased by 14% following higher consumption of the refined and processed pattern, while the odds of having a more metabolically healthy profile increased by 16% with increasing consumption of the healthy dietary pattern (adjusted analysis). Only two other studies, to our knowledge, were found that assessed diet and metabolic health with or without obesity [3,23]. In a sample of 45–85 year old men and women participating in National Health and Nutrition Examination Survey, 2007–2008 and 2009–2010, there were no significant differences in the Healthy Eating Index (2005) between those who were metabolically healthy and obese compared to those metabolically unhealthy and obese [23]. In a cross-sectional study of 6964 women in the US, food intake, as measured by a food frequency questionnaire, was not significantly different between obese women who were metabolically healthy or metabolically unhealthy [3]. That is, consumption of for example, fruits, vegetables, refined grains, sugar sweetened beverages, low/high fat dairy or take-away foods, were not different between those with or without metabolic syndrome (defined using both the American Heart Association/National Heart, Lung, and Blood Institute guidelines [24] and the homeostatic model assessment for insulin resistance [25,26]). That study however assessed single food group intakes rather than a complete dietary pattern, and the criteria for metabolic health between our study and that study was different. For example, our study used fasting plasma glucose status > 6.0 mmol/L or having current diagnosis of type 2 diabetes or unknown type, and systolic blood pressure ≥ 140 mmHg or diastolic blood pressure ≥ 90 mmHg, compared to that study using elevated blood pressure (≥ 130/≥ 85 mmHg) or drug therapy for hypertension, elevated glucose (≥ 5.6 mmol/L) or drug therapy for hyperglycemia. Given the limited results, drawing conclusions on the impact of metabolic health and obesity requires further investigation. However our results indicate that a healthier dietary pattern, consisting of wholegrains, fruit, legumes and low fat dairy is associated with a more metabolically healthy profile and these foods should be promoted to assist metabolic parameters.

We found no associations between metabolic health, as a continuous number of abnormalities, or between those considered metabolically healthy or metabolically unhealthy, and any of the dietary patterns. This contrasts with previous studies that have shown a Western dietary pattern was associated with incidence of metabolic syndrome over 9 years [27], metabolic abnormalities in 20,827 Chinese adults [28], as well as individual components of the metabolic syndrome [29,30]. A vegetarian dietary pattern was associated with a lower risk of metabolic syndrome in older adults in the US [31]; lower adherence to a Mediterranean dietary pattern was associated with greater number of metabolic syndrome parameters [32]; and men and women in the highest quartile of carbohydrate pattern (containing high intake of glutinous rice, fermented fish, chili paste, and bamboo shoots) had an 82% and 60% greater odds of having metabolic syndrome, respectively, than those in the lower quartiles of the carbohydrate pattern [33]. Similarly, a recent review by Calton *et al.*, highlighted the beneficial effects of the Mediterranean dietary pattern, the DASH diet, and the Nordic diet on metabolic syndrome components [12]. Additional large population studies are required to assist in drawing conclusions on the relationship between dietary patterns and metabolic health.

The pathophysiology of metabolically healthy obesity is likely the result of a number of underlying mechanisms and the interaction between genetic, environmental, and behavioral factors [34]. All of these factors have been shown to be related to fat distribution and accumulation, in addition to insulin resistance. Thus, the phenotype of those who are metabolically unhealthy and obese compared to those who are metabolically healthy and obese is different [35]. In particular, those who are metabolically unhealthy and obese have a greater proportion of subcutaneous fat [36] as well as increased markers of inflammation [37] compared to those who are metabolically unhealthy and obese. However, a recent meta-analysis of 20 studies (*n* = 359,137) reported a general non-significant relationship between metabolically healthy obesity and cardiovascular disease risk [15], indicating the metabolically healthy obese phenotype does not appear to confer greater risk for cardiovascular disease. These outcomes may lend support to our results in which we found no significant associations between number of metabolic abnormalities and dietary patterns, albeit an increased odds of having a more metabolically healthy profile with a healthier dietary pattern rather than a refined pattern.

Strengths of our study include the large population assessed and the rigorous data collection methods employed within the Australian Health Survey. Limitations to the study include the heterogeneous nature of the metabolic phenotype definition. However, we limited our criteria of metabolic phenotype to <3 abnormalities which several previous studies have also used [15], and our definition of metabolic health included waist circumference, rather than BMI as a marker of adiposity, as although BMI is useful in estimating body fatness, waist circumference provides an estimate of visceral adiposity and has been associated with insulin resistance, type 2 diabetes, and cardiovascular events [38]. Fasting glucose was also used to determine metabolic health, which has limitations in that in only provides a snap-shot of glucose regulation [39]; however the survey did not measure insulin resistance, thus we used the best marker available. Limitations of our study include its cross-sectional nature which does not assess change in diet and participants may have improved their diet if they had diagnosis of poor metabolic profile. That is, if participants were aware they had high blood pressure they may have reduced their sodium intake or increased their dairy intake; or if they had high cholesterol, they may have reduced their saturated fat and increased the unsaturated fat intake, hence improving diet quality. As such, capturing a single day of intake would have not have picked up these changes in diet. Further, using a 24-h recall, usual intake of an individual cannot be assessed, thus repeated 24-h recalls are needed to get population distributions of habitual intake. However, as the multiple pass system was used rather than a self-administered 24-h recall, this provides greater precision and provides the respondent with a number of occasions in which to reflect and accurately report on their intake by a trained researcher. Additionally, the survey was conducted over a year, thereby capturing seasonal intakes of different foods. Longitudinal studies would be ideal to capture any changes in diet and/or risk for developing adverse metabolic outcomes. The variance explained by our factors was lower than other previous studies [40,41,42,43], however the low variance could be partially explained by the fact that a number of participants were not eating some of the food groups thereby skewing the data and potentially indicating lower communality amongst the measured variables. Nevertheless, the Kaiser-Meyer-Olkin measure of sampling adequacy was greater than 0.5 indicating that factor analysis was appropriate for this data set and the food groups loading on the factors were varied and many were greater than the 0.25 cut-off value, suggesting our population had a varied diet that was, nevertheless, still specific to the identified factors. Finally, although we had some missing values for some of the metabolic markers, given the large sample size in this dataset, the number of missing values is unlikely to have influenced the results.

## 5. Conclusions

In summary, we identified that 20% of Australian adults aged ≥ 45 years were metabolically unhealthy and obese. Higher consumption of an unhealthier dietary pattern, characterised by refined and processed foods was associated with decreased likelihood of having a healthy metabolic and BMI profile, while the reverse was apparent for the healthy dietary pattern. Nutritional strategies addressing metabolic syndrome criteria and targeting obesity are recommended in order to improve metabolic phenotype and potential disease burden.

## Supplementary Files

Supplementary File 1
