# Supplementary material for: The Relationship between Dietary Patterns and Metabolic Health in a Representative Sample of Adult Australians
Source: Nutrients. 2015 Aug 5;7(8):6491–505. doi: 10.3390/nu7085295 (PMC4555134; doi:10.3390/nu7085295)
Supplement: Supplementary File 1 [file nutrients-07-05295-s001.docx]

**Supplementary File**

**Table S1.** Food groups included in the factor analysis (*n* = 39).

| **Food group used in the analysis** |
| --- |
| Whole grains |
| Refined grains |
| Red meats |
| Processed meat |
| Poultry |
| Meat-based mixed dishes |
| Take-away foods |
| Fried fish |
| Other fish |
| Fried potatoes |
| Potatoes |
| Yellow or red vegetables |
| Other vegetables |
| Legumes |
| Cruciferous vegetables |
| Leafy green vegetables |
| Tomato |
| Fresh fruit |
| Canned fruit |
| Dried fruit |
| Cakes, biscuits, sweet pastries |
| Low-fat dairy products |
| Full-fat dairy products |
| Soya products |
| Milk-based dishes |
| Confectionary |
| Added sugar |
| Crisps |
| Nuts |
| Sauces |
| Soups |
| Eggs |
| Tea, coffee |
| Soft drinks |
| Mineral water (plain) |
| Juice |
| Saturated spreads |
| Unsaturated spreads |
| Alcoholic drinks |

© 2015 by the authors; licensee MDPI, Basel, Switzerland. This article is an open access article distributed under the terms and conditions of the Creative Commons Attribution license (http://creativecommons.org/licenses/by/4.0).
